# Inferring Human Activity in Mobile Devices by Computing Multiple Contexts

**DOI:** 10.3390/s150921219

**Published:** 2015-08-28

**Authors:** Ruizhi Chen, Tianxing Chu, Keqiang Liu, Jingbin Liu, Yuwei Chen

**Affiliations:** 1 Conrad Blucher Institute for Surveying & Science, Texas A&M University Corpus Christi, Corpus Christi, TX 78412-5868, USA; E-Mails: tianxing.chu@tamucc.edu (T.C.); keqiang.liu@tamucc.edu (K.L.); 2 School of Environment Science and Spatial Informatics, China University of Mining and Technology, Xuzhou 221116, China; 3 Department of Remote Sensing and Photogrammetry, Finnish Geospatial Research Institute, National Land Survey of Finland, Masala 02431, Finland; E-Mails: jingbin.liu@nls.fi (J.L.); yuwei.chen@nls.fi (Y.C.)

**Keywords:** human activity recognition, mobile context computation, location awareness, smartphone positioning

## Abstract

This paper introduces a framework for inferring human activities in mobile devices by computing spatial contexts, temporal contexts, spatiotemporal contexts, and user contexts. A spatial context is a significant location that is defined as a geofence, which can be a node associated with a circle, or a polygon; a temporal context contains time-related information that can be e.g., a local time tag, a time difference between geographical locations, or a timespan; a spatiotemporal context is defined as a dwelling length at a particular spatial context; and a user context includes user-related information that can be the user’s mobility contexts, environmental contexts, psychological contexts or social contexts. Using the measurements of the built-in sensors and radio signals in mobile devices, we can snapshot a contextual tuple for every second including aforementioned contexts. Giving a contextual tuple, the framework evaluates the posteriori probability of each candidate activity in real-time using a Naïve Bayes classifier. A large dataset containing 710,436 contextual tuples has been recorded for one week from an experiment carried out at Texas A&M University Corpus Christi with three participants. The test results demonstrate that the multi-context solution significantly outperforms the spatial-context-only solution. A classification accuracy of 61.7% is achieved for the spatial-context-only solution, while 88.8% is achieved for the multi-context solution.

## 1. Introduction

Can a smartphone “think”? This is an interesting research question. Location-aware and context-aware applications are now appealing to mobile users, mobile industry and scientific communities. *Google now* is one of the smart applications publicly available nowadays with location-aware features. It calculates and pushes relevant information automatically to the mobile users based on his/her current location [1]. The user location is the trigger of the location-aware function. Context-aware applications are more complicated than location-aware applications because we need to look at the *whos*, *wheres*, *whens*, and *whats* (what is the user doing) to understand *why* the context is taking place [2]. In many cases, location-aware computing is not sufficient to understand concurrent activities occurring at the same location. More contexts are required in addition to the spatial context (location information). For example, a waitress may work in a coffee shop, while a customer may take a coffee break in the same place at the same time. We are unable to distinguish the waitress’ activity of “working” and the customer’s activity of “taking a coffee break” based on the location (where) and the time (when). However, the user mobility contexts are distinct between the waitress and the customer. The waitress walks around the coffee shop to serve different customers, while the customer stays mostly static at a coffee table to enjoy his/her coffee break. Furthermore, the spatiotemporal contexts are different as well. The waitress dwells much longer in the coffee shop than the customer. In general, a spatial-context-only approach works well in the situation that there is only one activity associated with a significant location. However, if multiple activities may take place at the same location, a multi-context approach is needed in order to exclusively identify the actual activity. This example explains our motivation to introduce a multi-context approach for inferring human activities.

Recognition of human activities plays a crucial role in smart context-aware applications. It will help the computer to understand what the user is doing under a particular circumstance. Although human activity recognition is a computationally demanding task, the rapid development of mobile computing capability in the last few years allows us to effectively achieve this goal in real time. Nowadays, the most common approaches for human activity recognition are based either on external sensing systems such as cameras in smart home environments, or on mobile sensing systems such as smartphones and wearable devices [3].

This paper introduces a framework for inferring human activities using smartphones. It is based on real-time computation of a series of contextual tuples. Each contextual tuple consists of:

(1) A spatial context, which is a geofence that can be a node associated with a circle, or a polygon;
(2) A temporal context, which can be the local time, a time difference between two geographical locations, or a timespan;
(3) A set of spatiotemporal contexts, which are location-dwelling lengths;
(4) A set of user contexts, which theoretically form a combination of user mobility contexts (e.g., static, walking, running, and driving), user environmental contexts (e.g., lighting condition, noise level, and weather conditions), user psychological contexts (e.g., levels of fatigue, excitement, and nervousness) and user social contexts (e.g., calling, texting/chatting, and using Apps). Within the scope of this study, only user mobility contexts are considered. However, the proposed framework is flexible for adopting other user contexts.

A Naïve Bayes classifier is employed in this study for inferring the significant activities. Preliminary results of this work have been published in [4]. Compared to our previous paper, the following significant contributions are addressed:

(1) A more comprehensive literature review is added;
(2) A new section describing our ubiquitous positioning solution is added. A dedicated experiment is conducted to assess the positioning performance in a typical indoor environment;
(3) The activity inference model has been massively upgraded by adapting multiple spatiotemporal contexts and user contexts into the contextual tuple;
(4) The live experiment has been enhanced with a week-long dataset including 710,437 labeled activities collected by different individuals.

The paper is organized as follows: Section 2 discusses the related research work; Section 3 presents the research methods including algorithms for positioning, motion detection, and activity inference; Section 4 discusses the field experiments and data analysis; and Section 5 concludes the paper and discusses future study.

## 2. Related Works

The location of a mobile user plays an essential role in enabling location-aware and context-aware applications, thus locating mobile users indoors/outdoors is considered as a prerequisite task. Smartphones are nowadays equipped with various built-in sensors and support multiple radio signals [5,6]. The sensors and radio signals offer us a unique opportunity to locate mobile users indoors/outdoors. For outdoor environments, a low-cost GPS receiver is capable of locating mobile users with an accuracy of about 5–10 m [7], which is adequate for many navigation and user tracking applications and a growing number of location-based services such as *Google now*. To locate mobile users indoors, measurements from other sensors (e.g., accelerometers, gyroscopes, and magnetometers) and radio signals (e.g., WiFi and Bluetooth) are required [8,9,10,11,12]. The positioning accuracy indoors varies depending on the level of supports from the ambient environments, e.g., the number of available WiFi access points and the spatial resolution of the radio map (or fingerprinting database). In any case, room-level positioning accuracy is achievable without any complex setup for indoor environments [13,14].

Although human activity recognition has been originally proposed since the late 1990s [3], the rapid improvements of computing and sensing capabilities in mobile devices have continuously underpinned the vitality due to its complexity and commercial potential for smart context-aware applications. 

Lara and Labrador [3] presented a summary of the current state of human activity recognition. As mentioned previously, there are two common sensing solutions for human activity recognition: the external sensing solution and the mobile sensing solution. Typical external sensors are cameras installed in smart environments, while typical mobile sensors are smartphone and wearable devices. 

Holder and Cook [15] presented a solution of recognizing and prompting human activities in smart home environments based on the events (e.g., door opening) recorded by the smart home system. Marszalek *et al*. [16] developed a solution for action and scene recognition simultaneously based on video clips. They have observed that action classification can be improved by 10% when including scene context in the classification procedure. Inversely, scene classification can also be improved by about 2% by using the action information in the classification.

For the mobile-based sensing solutions, accelerometers in smartphones have been successfully used for detecting user activities and motion patterns [8,17,18,19]. Pei *et al*. [20] developed a smartphone solution for human behavior/activity cognition. A model called LoMoCo (Location-Motion-Context) was developed in his study. The LoMoCo model utilized the user locations and motion states as inputs to infer human activities. A success rate of 92.9% was achieved in the study for inferring human behaviors using a least squares support vector machine classifier. Reddy *et al*. [21] used a mobile phone with a GPS receiver and an accelerometer to determine transportation modes of users. They achieved a success rate of 93.6% using a classifier of discrete hidden Markov model. Bancroft *et al*. [22] used a foot-mounted device equipped with a GPS receiver and an inertial measurement unit to determine motion-related activities including walking, running, biking, and moving in a vehicle. They achieved a classification error of less than 1%. Jin *et al*. [23] detected several motion states using an armband-mounted accelerometer and a “fuzzy inference system”. Liu *et al*. [24] proposed a solution to improve reciprocally the accuracy of indoor positioning and motion state recognition for each other, and further developed the geo-context concept that infers the users’ contexts by combing the location and motion states. Guinness [25] developed a solution of inferring daily mobility contexts based on (1) the built-in GPS receiver and the accelerometer in smartphones; and (2) location information of bus and train stations. He investigated a wide range of supervised learning techniques including decision tree, Bayesian network, logistic regression, artificial neural networks, support vector machine, and so forth. The classification success rates varied from 80.2% to 96.6%. The decision tree classifier with a success rate of 96.5% was found as the best classifier in terms of computation efficiency and classification accuracy.

## 3. Research Methods

We aim to develop a framework of inferring significant activity using mobile devices. Our solution is based on real-time computation of various contexts on a single mobile device. Group sensing and global sensing are out of the scope of this study.

The framework requires defining a set of significant activities to be inferred and a set of significant locations in advance. The mobile device computes all required contexts automatically in a real-time manner. Depending on the scope of the context-aware application, the definitions of the significant locations and activities can be different. However, an “undefined” significant location is always required to cover all other locations so that we can form a complete set of locations. Similarly, an “undefined” significant activity is also required to cover all other activities to form a complete set of significant activities.

Our approach looks at the *whens* (temporal context), the *wheres* (spatial context), and the *whats* (user and spatiotemporal contexts) to infer significant activities. The temporal contexts can be obtained directly from the system clock of the smartphone; the spatial contexts can be derived from our ubiquitous positioning solution implemented inside the smartphone; the spatiotemporal contexts can be obtained for each significant location by counting the cumulative dwelling seconds from the most recent time windows of, e.g., 60 s, and the user contexts can be derived e.g., from the measurements of motion sensors, ambient light sensors, microphones and so forth. A snapshot of a contextual tuple consists of a spatial context, a temporal context, a set of spatiotemporal contexts, and a set of user contexts. A snapshot of a contextual tuple is computed and recorded on a per-second basis. A significant activity is then inferred on a per-second basis by analyzing the most recent time series of the contextual tuples using a Naïve Bayes classifier.

The data processing procedure of the framework consists of the following steps:

(1) Raw data collection including measurements obtained from smartphone sensors and radio receivers;
(2) Real-time computation of the
  - temporal context by recording the local time,
  - spatial context by locating the user,
  - spatiotemporal contexts by counting the dwelling length at each significant location, and
  - user contexts by determining various user states including user motion states, user environmental states, user psychological states, and user social states;
(3) Inference of the significant activity.

For step 1, Android and iOS application program interfaces (APIs) offer sufficient functions and sample codes to access the measurements of the smartphone sensors and signal strengths of the radio signals. There is no need to address this issue in this paper. We next focus on real-time computation of the spatial contexts, spatiotemporal contexts, user contexts, and the inference of the significant activity.

### 3.1. Determination of the Spatial Contexts

The ultimate goal of spatial context determination is to identify the user’s current significant location, which is defined as a geofence that is, directly or indirectly, associated with one or more significant activities. Typical significant locations in our daily working life include e.g., offices, meeting rooms, dining halls, coffee stores, libraries, bus stops and so forth.

Satellite positioning has become a fairly mature technology and is adequate for determining the topological relationship between the user location and the predefined significant location. In challenging outdoor environments with reduced signal quality such as urban canyons, auxiliary techniques such as assisted GPS and dead reckoning can be employed to improve the positioning performance. On the other hand, indoor positioning does not benefit much from satellites as signals are affected by attenuation, reflection, and blockage. 

In order to achieve a real-time seamless indoor/outdoor positioning solution for deriving spatial contexts, we integrate measurements from a smartphone GPS receiver, motion sensors, inertial sensors and WiFi signals using an unscented Kalman filter (UKF). In this solution, if an activity takes place outdoors, the spatial context is purely determined by the GPS module, whereas measurements from motion sensors, inertial sensors and WiFi signals are utilized for indoor environments. The transition between indoor and outdoor environments is detected by measuring the GPS and WiFi signal strengths [20]. Since indoor positioning solution is usually more complex compared to the outdoor solution, we primarily discuss the indoor positioning solution in this paper.

Based on the received signal strength indication (RSSI), fingerprinting is a common approach for positioning indoors. A radio map (fingerprinting database) is needed for this approach. Once the radio signal patterns in the significant locations are mapped, the user can then be located at a reference point *P* that has the best match between its fingerprints and the observed RSSIs. Our matching algorithm is to find the reference point *P* in the database that has the shortest mean signal distance as follows:

(1) $$P=\underset{r\in \Omega }{\mathrm{argmin}}\left(\frac{1}{n}\sqrt{\sum_{i=1}^{n}w_{i}^{2}{\left({\bar{s}}_{o}^{i}-s_{r}^{i}\right)}^{2}}\right)$$

where *n* is the total number of matched access points that can be found from both observed RSSIs and fingerprints, *w_i_* is the weight of the RSSI measurement to the *i*th access point, $s_{r}^{i}$ is the *i*th RSSI fingerprint saved in the fingerprinting database, and ${\bar{s}}_{o}^{i}$ is the normalized observed RSSI measurement, which is estimated with:

(2) $$\begin{array}{l} {\bar{s}}_{o}^{i}=s_{o}^{i}-a\\ a=\frac{1}{n}\sum_{i=1}^{n}\left(s_{o}^{i}-s_{r}^{i}\right) \end{array}$$

where *a* is the bias between the observed and normalized RSSI measurements. *a* is introduced here to compensate common RSSI bias in order to utilize the same fingerprinting database for different types of smartphones. The weight $w_{i}$ is estimated with:

(3) $$w_{i}=\{\begin{array}{c} 1\;n\geq 10\\ n^{2}/100\;n<10 \end{array}$$

The weighting scheme is in favor of the solutions with a larger number of matched RSSI measurements.

Intermittent positioning outage occurs in the areas where the WiFi access points are not evenly distributed leading to poor signal reception. Therefore, pedestrian dead reckoning (PDR) is adopted to fill the gap between two consecutive WiFi fingerprinting updates. Equation (4) presents the PDR solution for propagating the planar coordinates:

(4) $$\begin{array}{l} e_{k}=e_{k-1}+s_{k-1}\Delta t\cdot \sin\alpha_{k-1}\\ n_{k}=n_{k-1}+s_{k-1}\Delta t\cdot \cos\alpha_{k-1} \end{array}$$

where $e_{k}$ and $n_{k}$ are east and north propagated coordinates given the known coordinates $e_{k-1}$ and $n_{k-1}$, **Δ***t* represents the time interval between epochs *k −* 1 and *k*, *s* and *α* depict the pedestrian speed and heading, respectively.

Smartphone compass provides absolute heading information by leveraging the accelerometer and magnetometer measurements, however, heading estimation can be severely contaminated wherever strong magnetic perturbation exists, particularly for indoor environments [5,19]. As a complementary sensor, gyroscopes generate angular rate without any adverse effect from magnetic field, yet they lack absolute orientation and are prone to drift significantly over time. In this study, we integrate these two sensors and take advantage of the floor plans to provide heading clues in indoor environments. On the other hand, user step frequency and walking speed can be estimated using accelerometer measurements. The detailed algorithm will be further discussed in Section 3.3.

To effectively integrate various data sources, an UKF filter is adopted to take into account both nonlinear dynamics and computational load on smartphone platforms. Based on Equation (4), we can define a user state vector as follows:

(5) $$x={\left[\begin{array}{ccccc} e & n & \dot{\alpha } & \alpha & s \end{array}\right]}^{T}$$

where $\dot{\alpha }$ depicts heading rate. The user dynamics can then be described as follows

(6) $$\begin{array}{l} e_{k}^{-}=e_{k-1}^{+}+s_{k-1}^{+}\cdot \Delta t\cdot \sin\alpha_{k-1}^{+}+w_{1}\\ n_{k}^{-}=n_{k-1}^{+}+s_{k-1}^{+}\cdot \Delta t\cdot \cos\alpha_{k-1}^{+}+w_{2}\\ {\dot{\alpha }}_{k}^{-}={\dot{\alpha }}_{k-1}^{+}+w_{3}\\ \alpha_{k}^{-}=\alpha_{k-1}^{+}+{\dot{\alpha }}_{k-1}^{+}\cdot \Delta t+w_{4}\\ s_{k}^{-}=s_{k-1}^{+}+w_{5} \end{array}$$

where $w_{i}\text{ (}i\in 1,\cdots ,5)$ are Gaussian system noises, the superscripts − and + stand for the prediction and the update of the state vector **x**, respectively. The measurement equation is as follows:

(7) zk=Hxk+vk     vk~N(0, Rk)

where measurement vector $z_{k}={\left[\begin{array}{ccccc} e_{WiFi} & n_{WiFi} & \dot{\alpha } & \alpha & s \end{array}\right]}^{T}$, measurement matrix **H** is 5 × 5 identity matrix, $v_{k}$ is a Gaussian measurement noise vector with zero mean and $R_{k}$ is the covariance matrix of the measurement vector.

During the time update phase, each sigma point is propagated via the nonlinear models. The mean and covariance of the forecast sigma points are computed with:

(8) $$\begin{array}{l} \chi_{i,k-1}\leftarrow x_{k-1}^{+},P_{k-1}\\ \chi_{i,k}=f(\chi_{i,k-1})z_{i,k}=h(\chi_{i,k-1})\\ x_{k}^{-}=\sum_{i=0}^{2L}W_{i}\chi_{i,k}\\ P_{k}^{-}={\sum_{i=0}^{2L}W_{i}\left\{\chi_{i,k}-x_{k}^{-}\right\}\left\{\chi_{i,k}-x_{k}^{-}\right\}}^{T}\\ z_{k}^{-}=\sum_{i=0}^{2L}W_{i}z_{i,k} \end{array}$$

$\chi_{i,k-1}$ contains a set of 2*L* + 1 sigma points calculated by unscented transformation; $W_{i}$ are the associated weights that can be determined according to [26]; *L* represents the dimension of the state vector (*L* = 5 in this study); $\chi_{i,k}$ and $z_{i,k}$ are predicted sigma points through the nonlinear models; $x_{k}^{-}$ and $z_{k}^{-}$ are the means of the corresponding predictions and $P_{k}^{-}$ is the covariance matrix of $x_{k}^{-}$. Once the WiFi and smartphone sensor measurements are available, the state vector is updated via

(9) $$\begin{array}{l} P_{z_{k}}={\sum_{i=0}^{2L}W_{i}\left\{z_{i,k}-z_{k}^{-}\right\}\left\{z_{i,k}-z_{k}^{-}\right\}}^{T}\\ P_{x_{k}z_{k}}={\sum_{i=0}^{2L}W_{i}\left\{\chi_{i,k}-x_{k}^{-}\right\}\left\{z_{i,k}-z_{k}^{-}\right\}}^{T}\\ K_{k}=P_{x_{k}z_{k}}P_{z_{k}}^{-1}\\ x_{k}^{+}=x_{k}^{-}+K_{k}{\mathrm{(z}}_{k}-z_{k}^{-})\\ P_{k}^{+}=P_{k}^{-}-K_{k}P_{z_{k}}K_{k}^{T} \end{array}$$

where $P_{z_{k}}$ is the innovation covariance matrix, $P_{x_{k}z_{k}}$ is the cross correlation matrix; $K_{k}$ is the gain matrix, $x_{k}^{+}$ is the updated state vector, and $P_{k}^{+}$ is the associated covariance matrix.

### 3.2. Determination of the Spatiotemporal Contexts

The main reason for us to adopt the spatiotemporal contexts as observables is that most human activities last for a certain period. For example, if you stay two seconds in a meeting room, we are unable to classify your current activity as “having a meeting” with high confidence. You might just stop by to request a signature from your boss who is participating a meeting. However, if you stay 5 min in the meeting room, we will be able to classify your current activity as “having a meeting” with a much higher confidence.

A spatiotemporal context (location-dwelling length) is a clear feature for most human activities. In this initial study, we evaluate the location-dwelling length by counting the cumulative dwelling seconds for each significant location from a 60-s sliding window of most recent time. The location-dwelling length is set to 1 if the total cumulative dwelling length is less than 5 s; to 2 if the dwelling length is between 6 and 15 s; and to 3 if the dwelling length is longer than 15 s as listed in Table 1. For every second, a location-dwelling length is evaluated for each significant location. Therefore, there will be *n* spatiotemporal contexts in each contextual tuple, where *n* is the number of significant locations.

**Table 1:** Definitions of the spatiotemporal context.

Observable	Description
1	*Dwelling length between 0 and 5 s*
2	*Dwelling length between 6 and 15 s*
3	*Dwelling length between 16 and 60 s*

### 3.3. Determination of User Contexts

A user context is a flexible parameter in a contextual tuple. Its general form is a combination of different categories of user related contexts, for example, including but not limited to:

(1) User mobility contexts such as motion patters;
(2) User psychological contexts such as levels of fatigue, excitement, nervousness, and depression;
(3) User environmental contexts such as ambient noise level, light intensity, temperature, and weather conditions;
(4) User social contexts such as calling, messaging/chatting, and using applications.

Table 2 summarizes the potential user contexts and their related observable sets. It serves as an example for showing the potential and complexity of the user contexts, rather than as a final list. More user contexts can be added to the framework without jeopardizing the theoretical fundamentals of the framework as long as the observables in the user contexts are independent. Depending on the complexity of the activities to be inferred, the user contexts might be complex. Adding more user contexts to the solution will increase the complexity of the probability model. However, it will increase the inferring capability of the framework. The powerful computational capability of smartphones allows us to explore a complex model for inferring complex activities.

The vector of user contexts is a complex but useful element in a contextual tuple because it increases the observability of the significant activities, leading to a better capability for inferring a wide range of significant activities. For example, the user motion patterns are observable from most human activities such as working in office (static), walking in a park (slow walking), and driving a car (fast moving). For other activities such as sleeping in a bedroom, both mobility context (static) and user environmental contexts (ambient noise level and light intensity) are observable. 

**Table 2:** Initial list of user contexts.

Category	User Context	Observable Set of Each User context
Mobility	Motion pattern	*static, slow walking, walking, fast moving*
Environment	Light intensity	*low, normal, high*
	Noise level	*low, normal, high*
	Temperature	*freeze, low, comfortable, high*
	Weather	*sunny, cloudy, raining, hazardous weather*
Psychology	Level of fatigue	*low, medium, high*
	Level of excitement	*low, medium, high*
	Level of nervousness	*low, medium, high*
	Level of depression	*low, medium, high*
Social	Social contexts	*calling, texting/chatting, using App*

In this initial study, only the following user mobility contexts are included: static, slow walking, walking, and fast moving. To determine the user mobility context, the user speed is determined based on a model developed by Chen *et al*. [8]. The user speed *v* is estimated with:

(10) $$\begin{array}{l} v=SL\cdot SF\\ SL=\left(0.7+a(H-1.75)+\frac{b(SF-1.79)H}{1.75}\right)c \end{array}$$

where *SL* is the step length, *SF* is the step frequency, *H* is the height of the pedestrian, and *a*, *b*, and *c* are the model coefficients with the values of 0.371, 0.227 and 1. The step frequency *SF* is detected in real time by analyzing the cyclic patterns of the accelerometer measurements. This model has a performance with the error of less than 3% in terms of travelled distance [8]. A pedestrian height of 1.75 m is adopted in this study instead of taking it from input of pedestrians. In the model, *c* is a personal factor that can be calibrated and adjusted on line if a known travelled distance is available. With this approach, user mobility context determination is an unsupervised process. The solution is rather stable and independent of the smartphone orientations. The user speed derived from Equation (10) is also applied to Equations (4)–(6) in Section 3.1.

### 3.4. Activity Inference

**Figure 1:**
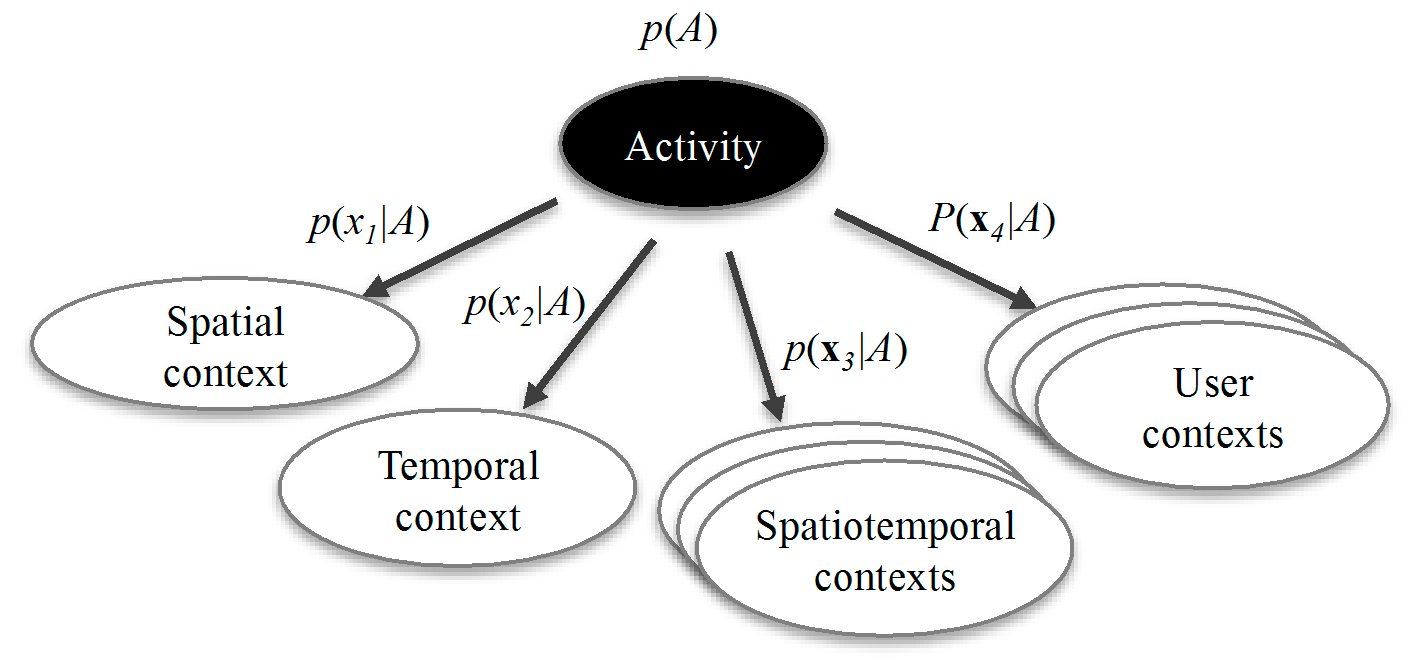
Structure of the Naïve Bayes classifier used for classifying significant activities.

Having obtained a time series of contextual tuples, the significant activity can then be inferred by using a Naïve Bayes classifier as shown in Figure 1. The conditional probability *p*(*A_k_*|**x**), which is the probability of activity *A_k_* given the observed contextual tuple **x** = [*a temporal context, a spatial context, a set of spatiotemporal contexts, a set of user contexts*], can be derived with:

(11) $$\begin{array}{l} p(A_{k}|x)=\frac{p(A_{k})p(x|A_{k})}{p(x)}\\ =\frac{p(A_{k})p(x|A_{k})}{\sum_{i}^{na}p(A_{i})p(x|A_{i})},\\ p(x|A_{k})=\prod_{j=1}^{d}p(x_{j}|A_{k}),\\ A=\mathrm{argmax}\;p(A_{k}|x),\;k=1,...,\;na,\;A\in \left\{A_{1},\cdots ,A_{na}\right\} \end{array}$$

where *na* is the total number of significant activities to be inferred, *d* is the dimension of a contextual tuple, which is dependent on the number of the spatiotemporal contexts and the user contexts included in the model.

In order to estimate the conditional probability *p*(*A_k_*|**x**), we need to know the probability model **P** = (**A**, **B**), which includes:

- The probability distributions of the significant activities including an undefined activity: **A** = [*p(A_1_), p(A_2_), ..*., *P(A_na_)*], with $\sum_{i=1}^{na}P(A_{i})=1$,
- The observation probability matrix **B** for all *p*(*x_i_|A_k_*), where *i = 1*,…,*d*, and *k =1*,...,*na*. **B** is a *d × na* dimensional matrix of probability density functions (PDFs). Each PDF is a histogram with various bins depending on the size of the sample set of the corresponding observable, e.g. the size of the sample set of a user mobility context [*static*, *slow walking*, *walking*, *fast moving*] is four. For each PDF, we have $\sum_{j=1}^{nb}P_{j}=1$, where *P_j_* is the probability of the *j*th element in the sample set, and *nb* is the size of the sample set.

The traditional way of obtaining the probability model **P** = (**A**, **B**) is through training. It is a tedious task, as it requires a large training dataset with labeled classes in order to obtain an accurate model. A dedicated machine learning algorithm is needed in this scenario. Model training is supported in our framework using e.g., a hidden Markov model. We will address this topic in a separate paper.

**Figure 2:**
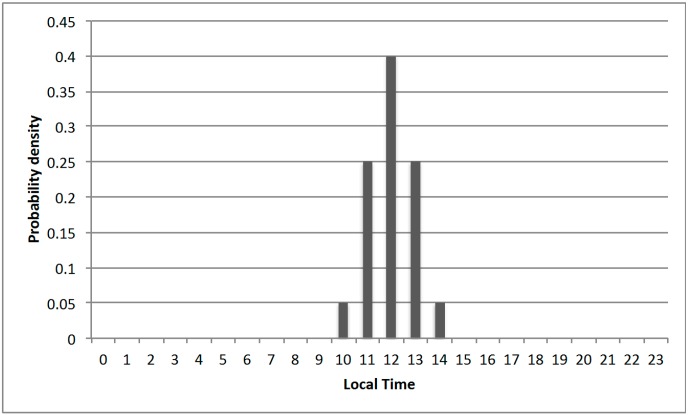
An empirical probability distribution of the observable local time given the activity of “having a lunch”.

Another approach to obtain the probability model is to adopt an empirical model based on existing knowledge. For example, people normally have lunch between 11:00 a.m. and 1:00 p.m. Thus, the probability distribution of the observable local time (temporal context) given the activity of “having a lunch” can be described by a histogram shown in Figure 2. In practice, the empirical model can be adopted as an initial model. This initial model can be improved with labeled or inferred classes by a self-learning process. 

**Figure 3:**
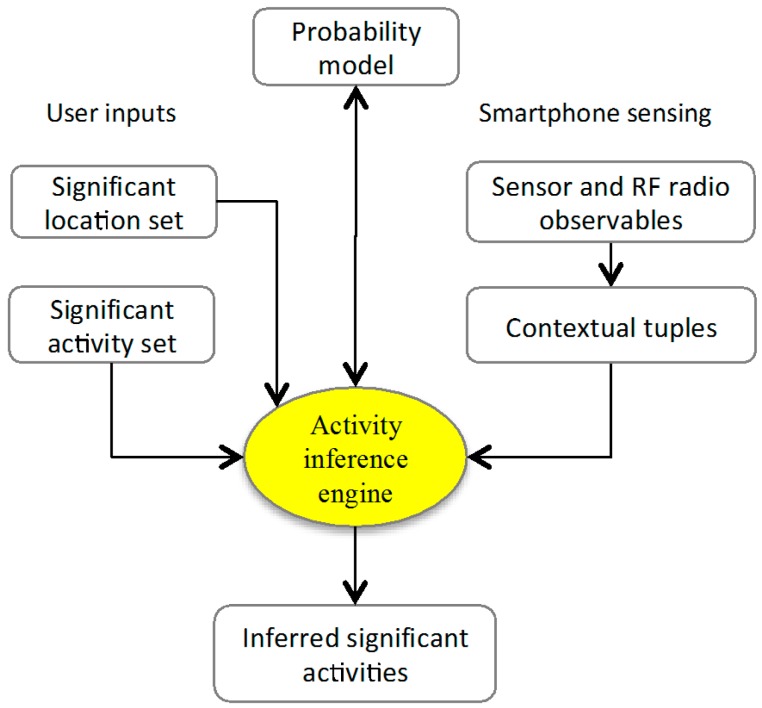
Diagram of the procedure for inferring significant activities. The probability model can be either from user input (empirical model) or from training process using labeled activities.

Figure 3 shows the diagram of the procedure for inferring significant activities. It mainly consists of two parts: user input and mobile sensing. The part of user input includes the definitions of the significant locations and activities, while the mobile sensing part includes the real-time computation of the contextual tuples. The probability model can be derived either by user input (empirical model) or by mobile sensing (model trained using labeled activities).

This paper focuses on introducing the multi-context approach for inferring human activity, rather than assessing the performances of various machine learning based activity inference algorithms. The Naïve Bayes classifier is chosen to demonstrate the functionality of our activity inference engine because it is

(1) A computationally efficient approach that can be implemented in smartphones;
(2) Flexible in supporting incremental learning;
(3) Insensitive to irrelevant features.

## 4. Experiments and Data Analysis

Two experiments were conducted for (1) evaluating the accuracy of the indoor positioning algorithm; and (2) assessing the performance of the framework of significant activity inference.

### 4.1. Evaluation of the Indoor Positioning Accuracy

Significant locations play an essential role in our framework. For outdoor environments, the GPS receiver in the smartphone provides positions with sufficient accuracy for our purpose. However, most human daily activities occur indoors. Therefore, the performance of our indoor positioning solution is critical. It has direct impact to the final success rate of activity inference. For this reason, we conducted a dedicated experiment to verify the positioning accuracy of our indoor positioning algorithm described in Section 3.1. 

The experiment was carried out in the building of Natural Resources Center (NRC) at Texas A&M University Corpus Christi. The NRC building is a three-story facility with no GPS reception indoors. The experiment setup was on the first floor of the NRC building where WiFi access points are installed unevenly. Furthermore, the test environment has strong magnetic perturbation. Our positioning software running in a Samsung Galaxy Note 3 smartphone was tested against the ground truth derived from the NovAtel SPAN-IGM-S1 GNSS/INS system. The detailed specifications of the SPAN-IGM-S1 system are available at [27].

**Figure 4:**
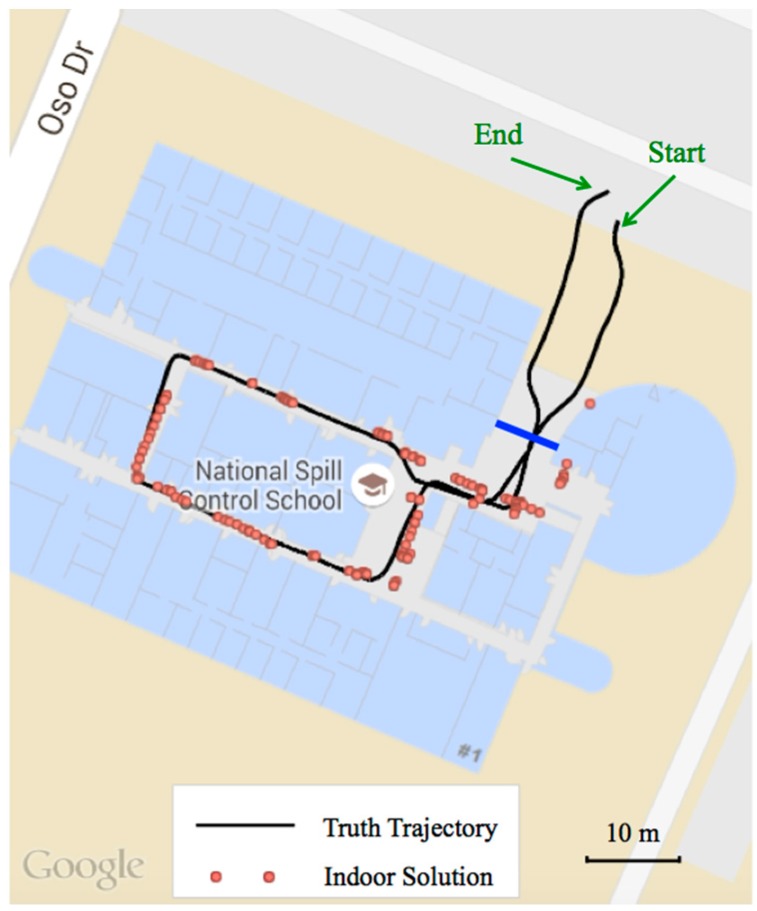
The trajectories of the indoor positioning solution with a Samsung Galaxy Note 3 smartphone and the ground truth generated by the NovAtel SPAN-IGM-S1 system.

Figure 4 illustrates the indoor positioning trajectories of the smartphone-based solution and the ground truth. The red dots represent the results obtained indoors from our positioning algorithm presented in Section 3.1 while the smoothed black trace shows the ground truth. The blue solid line indicates the building entrance that separates the indoor and outdoor environments. Outdoor positioning results obtained by the smartphone are not shown as we mainly focus on the indoor environment. To avoid excessive IMU drift in the NovAtel SPAN system, the experiment started and ended outdoors in an open sky condition as indicated in the figure. It can be noticed in the figure that for most epochs the smartphone-based results are aligned well with the ground truth.

**Figure 5:**
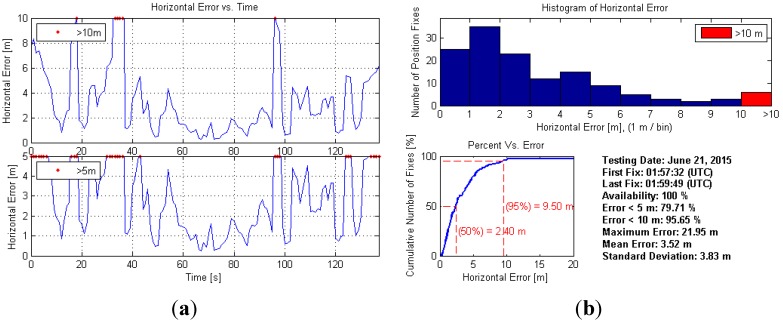
Horizontal positioning error statistics. (**a**) illustrates the horizontal positioning error in two different zoom levels and (**b**) shows the histogram, cumulative distribution curve and other related statistics.

Figure 5 shows the detailed indoor positioning error statistics: the left side illustrates the horizontal positioning error in two different zoom levels, while the right side shows the histogram, cumulative distribution curve and other related statistics. It can be concluded that meter-level horizontal accuracy is achieved indoors for most epochs using the proposed algorithm. However, instantaneous error spikes exceeding 10 m still occur occasionally due to the jumpy and noisy nature of wireless positioning indoors. By using an elaborated clustering strategy, the overall accuracy is expected to further improve. Moreover, it is also found that near the building door (*i.e*., the indoor/outdoor transitional area) the positioning accuracy is relatively poor according to Figure 4 and both ends of the subplots on the left side of Figure 5. The major reason is the switching between indoor/outdoor navigation methods, *i.e*., between the GPS solution and the WiFi/PDR solution.

### 4.2. Assessment of the Activity Inference

To evaluate the performance of the Naïve Bayes classifier implemented in this framework, a test environment was setup at the campus of Texas A&M University Corpus Christi. We have conducted an experiment with three participants including a professor, a post-doc research associate and a PhD student for an entire week. 

As shown in Figure 3, we need to define the significant locations and significant activities before carrying out the experiment. In our experiment, we defined seven significant locations and seven significant activities. A prototype of the framework was developed on the Android platform for both real-time inference and data logging. Two Samsung Galaxy S4 and one Samsung Galaxy Note 3 smartphones were used during the experiment.

#### 4.2.1. Significant Locations

**Figure 6:**
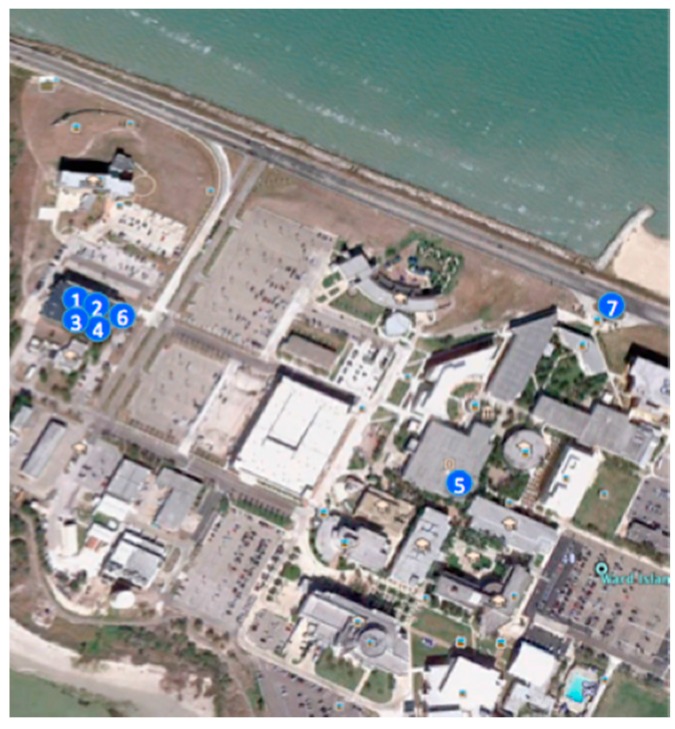
The significant locations used in the experiment. The number tags of these locations are defined in Table 3.

**Figure 7:**
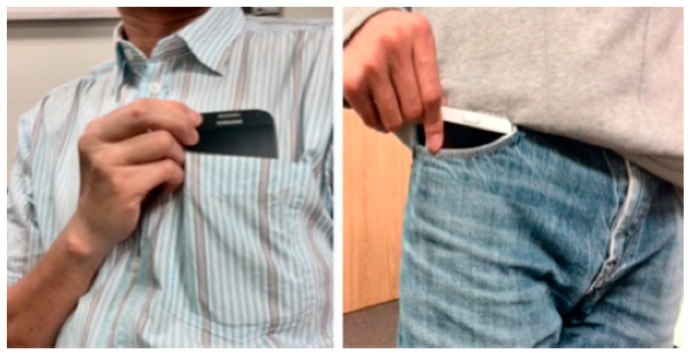
Poses of the mobile devices during data logging.

Table 3 lists all significant locations defined for the test environment on campus. A significant location is not a point but rather a geofence, which is defined as a non-overlapped polygon. Whenever a mobile device is located within a polygon, the corresponding significant location is then identified; otherwise, the location ID of “undefined location” is recorded. Figure 6 shows the physical locations of these significant locations. They are located in three clusters: the first cluster contains the “Office”, the “Meeting Room” and the “Kitchen” that locate closely to each other inside the same suite of our laboratory; the second cluster contains the “Coffee Break Area” and the “Classroom” that locate in the same building but on different floors; the last cluster contains the “Library” and the “Bus Stop” that locate far away from other significant locations. The closely located significant locations in the first cluster (e.g., the meeting room is next door to two of our offices) bring some difficulties to identify the significant locations precisely, especially when we put the devices in our pockets as shown in Figure 7 during data logging. The positioning errors have direct impacts on two elements in the contextual tuple: the significant location and the location-dwelling lengths.

**Table 3:** The list of significant locations.

Location-ID	Description
1	Office
2	Meeting room
3	Kitchen
4	Coffee Break Area
5	Library
6	Classroom
7	Bus Stop
8	Undefined Location

#### 4.2.2. Significant Activities

The experiment consists of eight activities: seven significant activities and one undefined activity. The undefined activity covers all activities other than the pre-defined ones. Table 4 lists the definition of these activities and the corresponding prior probabilities.

In terms of “Working”, we include the activities such as working in office and some miscellaneous short-term activities such as fetching papers from the printers, fetching a cup of water from kitchen, and going to the restroom. We labeled the activities in common sense to fit our real daily life (and easy for us to label our activities for an entire week). The reason for assigning “Other activities” a highest probability is because all activities after work belong to “Other activities”.

**Table 4:** Prior probability of each significant activity.

Activity-ID	Description	Probability
1	Working	0.3333
2	Having a meeting	0.0208
3	Having a lunch	0.0417
4	Taking a coffee break	0.0208
5	Visiting library	0.0208
6	Taking a class	0.0573
7	Waiting for bus	0.0053
8	Other activities (undefined activities)	0.5000
	**Total**	**1.0000**

#### 4.2.3. Data Logging

We utilized a self-developed mobile App running on Android platform to log the data. It determines and records the contextual tuples in real time on a per-second basis including the following functions:

(1) Recording the local time tag;
(2) Locating the mobile users ubiquitously indoors/outdoors using GPS, built-in sensors, and WiFi signals;
(3) Determining the location-dwelling length for each significant location;
(4) Determining the user mobility context using the built-in accelerometer;
(5) Logging the time series of the contextual tuples and labeling the real activity for each second.

The first element in the contextual tuple is the local time, which is a temporal context. It is obtained directly from the system clock of the smartphone.

The second element in the contextual tuple is the significant location, which is a spatial context. It is derived from the ubiquitous positioning module described in Section 3.1. As shown in Figure 7, we put the smartphones in our pockets during data logging in order to keep the devices with us all the time. This is not a favorable situation for positioning because our bodies block/attenuate a large part of the WiFi signals leading to a lower positioning accuracy compared to the case when holding the device in hand. However, this will help track our activities in a realistic way.

The third element in the contextual tuple is a set of location-dwelling lengths, which are spatiotemporal contexts. They are cumulative numbers of dwelling seconds at significant locations counted within the most recent 60-s sliding window. In this study, three observables for a location-dwelling length are defined and coded as listed in Table 1.

The last element in the contextual tuple is a set of user contexts that combine different user states. User contexts are complex as shown in Table 2. In this initial study, only user mobility contexts consisting of four states are considered as defined in Table 5.

A large dataset containing 710,436 contextual tuples was recorded by three participants with the Samsung smartphones as mentioned previously.

### 4.3. Data Analysis

Three solutions were analyzed: an unsupervised solution, a supervised solution, and a spatial-context-only solution, which was a supervised solution purely based on spatial contexts. From the point view of probability models, solution 1 adopts an empirical model without a model training phase; solution 2 utilizes a model trained with multiple contexts, and solution 3 applies a probability model trained with the spatial contexts only. From the point view of context diversity, solutions 1 & 2 are multi-context approaches, while solution 3 is a spatial-context-only approach. Table 6 lists the details of all solutions. The dataset was analyzed with Weka software [28].

The one-week dataset of each participant was analyzed independently with all three solutions. The entire dataset was divided into 10 pieces. A 10-fold cross-validation procedure was applied to solutions 2 and 3 to assess their performances. For the unsupervised solution, all labeled activities were used to assess the performance. Furthermore, the same empirical model was applied to all three participants.

Two comparisons were carried out as shown in Table 6. The first one was an unsupervised vs supervised comparison between solutions 1 and 2, while the second one was a multi-context *vs*. spatial-context-only comparison between solutions 2 and 3.

**Table 5:** Definition of the user mobility contexts.

ID	Description
1	static, speed <= 0.1 m/s
2	slow walking, 0.1 m/s < speed <= 0.7 m/s (less than one step per second)
3	walking, 0.7 m/s < speed <= 1.4 m/s (1–2 steps per second)
4	fast moving, speed > 1.4 m/s (more then 2 steps per second, or driving)

**Table 6:** Definitions of three different processing solutions.

Solution#	Supervised/Unsupervised	Multi-context/Spatial-Context-Only	Probability Model
1	Unsupervised ^a^	Multi-context	Empirical
2	Supervised ^a^	Multi-context ^b^	Trained with multiple contexts
3	Supervised	Spatial-context-only ^b^	Trained with spatial context only

^a^ Comparison group 1; ^b^ Comparison group 2.

#### 4.3.1. Comparison between the Supervised and Unsupervised Solutions

Table 7 shows the classification accuracy of the supervised and unsupervised solutions. It is obvious that the supervised solution significantly outperforms the unsupervised solution for all participants. The average success rate of the supervised solution is 88.8%, while that for the unsupervised solution is 58.0%. The main reason for the low performance of the unsupervised solution is its approximation of the probability model. Besides, we applied the same model to three different participants who have different life patterns (someone comes to work at 8:00 a.m., while others at 9:30 a.m.). It is obvious that the empirical model fits best with the first participant but worst for the second participant. On the other hand, the classification accuracies of all participants using the supervised solution are comparable because the probability model for each participant was derived from the dataset itself. With this comparison, we can conclude that a proper empirical model can be used to recognize human activities at a success rate of about 60%. In order to obtain higher classification accuracy, a model training process for each individual is needed.

#### 4.3.2. Comparison between the Multi-Context and Spatial-Context-Only Solutions

Comparison between the multi-context and spatial-context-only solutions was performed in order to assess the benefits of introducing multiple contexts to our inference framework. The multi-context solution is based on the multiple contexts including the spatial contexts, the temporal contexts, the spatiotemporal contexts and the user contexts, while the spatial-context-only solution includes only the spatial context (significant location). As listed in Table 6, both solutions are supervised solutions. Table 8 lists the performances of the multi-context and spatial-context-only solutions for all three participants.

**Table 7:** Classification accuracy of the supervised and unsupervised solutions for three participants with one-week datasets.

Participant	Solutions		Number of Labeled Activities
	Unsupervised (Solution 1)	Supervised (Solution 2)	
1	66.5%	88.9%	237,085
2	50.3%	87.9%	211,834
3	57.2%	89.6%	261,517
Mean	58.0%	88.8%	236,812

**Table 8:** Classification accuracy of the multi-context and spatial-context-only solutions for three participants with one-week datasets.

Participant	Solutions		Number of Labeled Activities
	Spatial-Context-Only (Solution 3)	Multi-Context (Solution 2)	
1	64.0%	88.9%	237,085
2	65.7%	87.9%	211,834
3	55.4%	89.6%	261,517
Mean	61.7%	88.8%	236,812

It is obvious that the multi-context solution significantly outperforms the spatial-context-only solution. The average success rate of the spatial-context-only solution is 61.7%, while that for the multi-context solution is 88.8%. The main reason for the evident improvement in the multi-context solution is the additional information derived from other contexts, which increases the observability of the significant activities. With this comparison, we can conclude that the introduction of multiple contexts to the solution increases the observability of the significant activities leading to a significant improvement of the success rates of activity inference.

## 5. Conclusions and Future Works

This paper presented a framework for inferring significant activities in mobile devices. The framework takes the advantage of multiple contexts including the spatial contexts, the temporal contexts, the spatiotemporal contexts, and the user contexts. It is a solution beyond the scope of location awareness by introducing more distinct contextual features and increasing the observability to the significant activities. The framework was tested in a typical campus environment at Texas A&M University Corpus Christi. We inferred seven typical on-campus activities including working, having a meeting, having a lunch, taking a coffee break, visiting the library, taking a class, and waiting for the bus. The experiment was conducted for an entire week by three participants including a professor, a post-doc research associate, and a PhD student. After analyzing 710,436 labeled activities, the results demonstrate that the multi-context approach outperforms the spatial-context-only approach significantly. The success rate of the spatial-context-only solution is 61.7%, while that for the multi-context solution is 88.8%.

Regarding to future work, we will adopt more user contexts such as audio signals, ambient light intensity, and temperature information to enhance the success rates of activity inference.
